# Biomaterials: A potential pathway to healing chronic wounds?

**DOI:** 10.1111/exd.13290

**Published:** 2017-02-14

**Authors:** Mara A. Pop, Benjamin D. Almquist

**Affiliations:** ^1^ Department of Bioengineering Imperial College London London UK

**Keywords:** biomaterials, chronic wound, diabetic ulcer, tissue repair, wound healing

## Abstract

Chronic dermal wounds are a devastating problem, which disproportionally affect individuals with conditions such as diabetes, paralysis, or simply old age. These wounds are extremely challenging to treat due to a heterogeneous combination of causative factors, creating a substantial burden on healthcare systems worldwide. Despite their large impact, there is currently a startling lack of options for effectively treating the underlying biological changes that occur within the wounds. Biomaterials possess an enticing ability to provide new comprehensive approaches to healing these devastating wounds; advanced wound dressings are now being developed that enable the ability to coordinate temporal delivery of multiple therapeutics, protect sensitive biologics from degradation, and provide supportive matrices that encourage the growth of tissue. This positions biomaterials as a potential “conductor” of wound repair, allowing them to simultaneously address numerous barriers to healing, and in turn providing a promising pathway to innovative new technologies for driving successful healing.

## 1 Chronic Skin Wounds are a Major Burden on Individuals and Society

Chronic skin ulcerations such as venous, decubitus or diabetic ulcers have a large impact on societies in both developed and developing countries around the world.^1^ In the UK, they affect an estimated 1% of the total UK adult population, and as high as 5% of the UK population over 65.^[s1]^ These devastating wounds have an enormous negative impact on an individual's quality of life, leading to loss of mobility and sleep deprivation and contributing to increased risk of amputation, anxiety, and depression.^[s2‐s6]^ Looking solely at diabetic foot ulcers (DFU), diabetic individuals possess a 23‐fold increase in the rate of amputation following ulceration compared with non‐diabetics, with up to 85% of amputations preceded by DFUs.^[s6]^ Moreover, the 5‐year mortality rates associated with DFUs or DFU‐related amputations have been found to be as high as or higher than those of breast and prostate cancer.^[s7]^ With an increasing frequency of diabetes and obesity, along with an ageing population, these values are only expected to rise without any further innovations for treatment.

## 2 Biomaterials Enable Novel Therapeutic Approaches

Despite the large impact of these wounds, there are surprisingly few options available for treating them that take advantage of recent insights from the laboratory. While conventional strategies such as debridement, negative pressure therapy, and offloading orthotics can lead to successful wound closure in many patients,^[s8]^ these treatments are ineffective for a significant number of individuals. Many times this resistance to treatment may stem from underlying biological changes that affect the ability of cells within the skin to properly carry out the process of tissue repair;^[s9]^ cells isolated from DFUs have been shown to display differences in their response to growth factors,^[s10]^ proliferation rates,^[s11]^ composition of the extracellular matrix (ECM) they deposit,^[s12]^ levels of proteases produced,^[s13,s14]^ and regulatory RNA molecules such as microRNAs.^[s15]^

**Figure 1:**
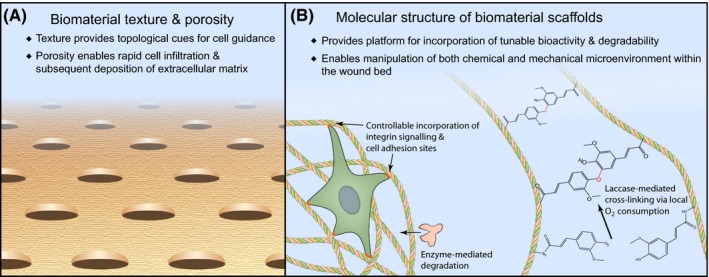
(A) The texture, topology, and porosity of biomaterials can be used to facilitate rapid cell infiltration and subsequent matrix deposition. (B) Manipulating the molecular structure of biomaterials allows for controllable incorporation of cell adhesion sites, degradation mechanisms, mechanical properties, and regulatory mechanisms for manipulating the local microenvironment

The ability to correct these biological abnormalities within the wound microenvironment has been actively explored as a key strategy for developing new therapeutic options. Unfortunately, the translation of pharmaceutical‐based approaches from the bench to bedside over the past two decades has been a splendid graveyard of failures. While this may seem dire in terms of future prospects, the field of tissue engineering and biomaterials has slowly played a more active and important role towards achieving translational success.^[^ 2 ^,s16‐s24]^ Significant emphasis has been put on the development of bioengineered skin replacements, such as decellularised matrices, living skin constructs and degradable polymeric scaffolds that mimic multiple features of the native ECM and allow modulation of the matrix within the wound bed.^[s25‐s28]^ Such scaffolds, whether natural or synthetic, have porous microstructures tagged with cell‐binding sites to support the infiltration and adhesion of cells, allow diffusion of nutrients and metabolites, have controllable degradation mechanisms, and can act as reservoirs to retain bioactive agents that stimulate innate tissue repair mechanisms^[^ 2 ^,s24,s29]^ (Figure 1). In this way, they act as a support to the native wound microenvironment to regulate cell behaviour, such as the proliferation, migration, and differentiation of cells within the wound, providing instructive cues that are crucial for efficient wound closure.

When aiming to synthesise biomaterial scaffolds, there are many avenues that are available to pick from depending on the required functionality of the scaffold. For instance, in one study ECM‐mimicking scaffolds have been developed to encourage cell migration.^3^ These microporous scaffolds are electrospun from collagen I (col) and poly (ε‐caprolactone) (PCL) in a 70:30 ratio, with pores of 160 μm diameter subsequently being introduced mechanically throughout the thickness of the scaffold. This architecture creates an optimal mesh that fosters fibroblast infiltration, attachment, and proliferation in vitro. The authors demonstrated that primary human fibroblasts isolated from neonatal foreskin migrate into the scaffolds and secrete native ECM molecules, such as collagen and fibronectin, while also remodelling the scaffold to mimic the normal dermal matrix. When implanted into full‐thickness rat skin wounds, these 70:30 col/PCL scaffolds stimulated more rapid and effective wound healing compared with scaffolds lacking micropores or sham wounds.

In other work, researchers demonstrated that a dextran‐based hydrogel scaffold consisting of UV‐cross‐linked dextran‐allyl isocyanate‐ethylamine and polyethylene glycol diacrylate polymers are able to promote regenerative healing in a third‐degree mouse burn wound model.^4^ This hydrogel, with no additional growth factors, cytokines, or cells, facilitates early infiltration by inflammatory cells that leads to rapid degradation of the hydrogel, in turn promoting significant neovascularisation by day 7, followed by complete skin regeneration at 3 weeks postwounding. When recently tested in a porcine burn wound model, the same hydrogel promoted tissue regeneration through fast neovascularisation, formation of ECM‐rich granulation tissue, and enhanced re‐epithelialisation of the wound area compared with control dressings.^[s30]^ Other work by the group has given rise to a hypoxia‐inducible hydrogel that guides vascular morphogenesis in vitro and in vivo by establishing precise oxygen gradients generated within the hydrogel.^5^ Although not currently used for the treatment of chronic wounds, biomaterials that can manipulate local oxygen concentration within ischaemic diabetic and venous ulcers are a potentially interesting approach for promoting wound resolution.

**Figure 2:**
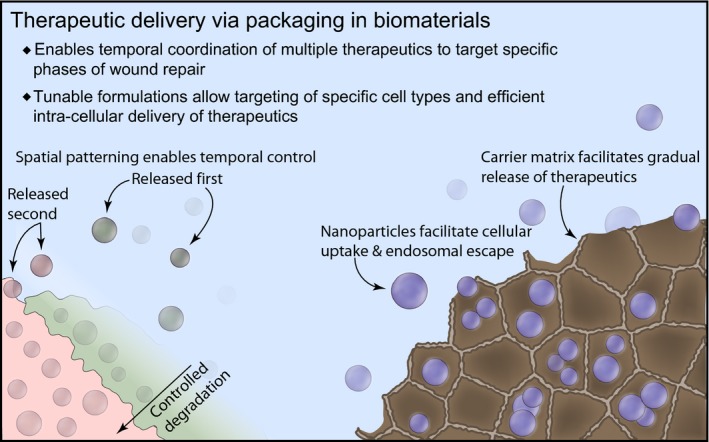
Biomaterials present a flexible approach to controlled delivery of therapeutics to local wound sites. They enable temporal coordination of multiple synergistic therapeutics, provide protection for sensitive biologics from the hostile wound environment, and facilitate efficient intra‐cellular delivery

While these examples provide options for creating matrices that manipulate the local wound environment and provide a scaffold for infiltrating cells, it may be necessary to also deliver sensitive biologics, such as growth factors or nucleic acids, to fully address the biological deficiencies observed in chronic wounds (Figure 2). These biological agents require complex delivery mechanisms that protect them from degradation by enzymes in the hostile wound environment, control the rate of therapeutic release to present an extended physiologically relevant dosage, and facilitate efficient localised therapy at the appropriate site of action. Assuming these hurdles are overcome, the challenge of treating wounds that display such heterogeneous changes using a *single* therapeutic is still a daunting prospect. These wounds likely require advanced wound dressings that can coordinate the temporal delivery of multiple therapeutics into the wound bed. In doing so, each therapeutic can build on the impact of preceding ones and further synergise with each other for improved efficacy.

However, traditional delivery platforms such as creams (e.g. becaplermin), sprays (e.g. trafermin) and injections (e.g. Heberprot‐P) are unlikely to provide the control necessary to fully exploit this strategy. Using these methods does not enable the ability to easily tune the spatiotemporal delivery pattern, resulting in large initial bolus releases and high‐dosage deliveries that may lead to serious side effects, while also requiring a high level of patient compliance that many times presents a significant challenge when treating chronic wounds.^[s31,s32]^

To overcome some of these barriers, we have recently developed a self‐assembled coating for wound dressings that enables sustained release of multiple active growth factors while allowing independent modulation of their individual release kinetics.^6^ In this work, we incorporated VEGF‐165 and PDGF‐BB onto woven nylon wound dressings using the layer‐by‐layer (LbL)^[s33]^ process. Experiments in db/db mice demonstrated increased levels of both angiogenesis and formation of granulation tissue with the dressings. This was accomplished using a total growth factor dose of approximately 150 ng, an amount 300 times lower than previously used for the FDA‐approved becaplermin gel.^[s34]^ Further to this, we demonstrated that the LbL technique is able to create wound dressings that facilitate localised release of siRNA targeting MMP‐9 (siMMP‐9) in db/db mice.^7^ This strategy led to a reduction in MMP‐9 expression and activity after 2 weeks of over 75% and 55%, respectively, resulting in dramatic improvements in the rate of granulation tissue formation and re‐epithelialisation.

While these two examples demonstrate the flexibility of a technique such as LbL, there are many other strategies that have been developed to enable efficient delivery of biologics. For instance, recent research has demonstrated that sustained siRNA delivery to skin wounds is possible by packaging siRNA molecules within pH‐responsive polymer nanoparticles composed of 2‐dimethylaminoethyl methacrylate, which are then incorporated in biodegradable injectable polyurethane scaffolds.^8^ The nanoparticles protect the siRNA from degradation by nucleases and ensure the siRNA is delivered once it enters an acidifying endosome (pH 5.8), instead of being released in the extracellular milieu. In this study, the delivery platform promoted angiogenesis in a balb/c mouse wound model through sustained and controlled knockdown of the enzyme prolyl hydroxylase 2, PHD2. This resulted in a significant increase in HIF1α‐mediated transcription of the pro‐angiogenic genes VEGF and FGF‐2, in turn leading to a 280% increase in vessel area at day 33. Recently, the same group has demonstrated that PHD2 knockdown via ROS‐degradable scaffolds also improves wound healing in diabetic mice.^9^ These examples together illustrate how carefully designed biomaterial‐based platforms have an enormous potential to efficiently target biological changes in chronic wounds.

## 3 The Pathway Forward

There are many interesting biological targets that need further exploration and validation in the context of chronic wound healing, as exciting new developments are constantly being made in this area. Recent findings have elucidated an important role of Ephrin‐B1 in regulating epithelial cell‐cell junctions during re‐epithelialisation, with clinical non‐healing chronic wounds displaying strong upregulation.^[s35]^ In other research, it has been shown that persistent PKCδ expression inhibits insulin signalling in fibroblasts from type 1 diabetic wounds, with siRNA knockdown of PKCδ resulting in increased levels of p‐AKT, VEGF expression and rate of wound healing in vivo.^[s36]^ Finally, sustained release of stromal cell‐derived factor‐1 (SDF‐1) from an antioxidant thermoresponsive hydrogel has been shown to significantly enhance formation of granulation tissue and epithelial maturation in non‐healing DFUs.^[s37]^

How these new insights can be integrated into a comprehensive therapeutic strategy that builds on extensive prior findings is still unknown; such a strategy will likely require a combination of proteins, nucleic acids, cells, and polymeric scaffolds that act together in a concerted manner to synergistically rewire the local wound environment to drive healing. This is where biomaterials enter the picture as an enabling technology that can simultaneously address differential delivery needs for each therapeutic (intra‐cellular, extracellular), optimise dosing schedules, and provide a supportive extracellular matrix for the wound microenvironment. These strategies for treating chronic wounds will then likely need to be paired with the development of various biomarkers that can effectively stratify patient populations into treatable cohorts. For instance, patient stratification based on macrophage gene expression,^[s38]^ or potentially by using non‐invasive imaging of wounds^[s39]^ to identify wounds that are most likely to benefit from certain treatment strategies, is crucial for maximising the success of advanced therapies.

However, despite these stimulating advances in the field of biomaterials for wound healing applications, hurdles that still need to be addressed include the manufacturability of the materials as the level of complexity increases, along with the toxicity and biocompatibility of these materials with the host tissue. Care must be taken when designing degradable hydrogels for tissue repair, as these gels can form toxic degradation products (e.g., carboxylic acid products derived from ester‐based degradation mechanisms). Such products have the ability to raise the local acidity of the surrounding tissue and trigger a pronounced inflammatory response over time;^[s40]^ ideally, biodegradable hydrogels will create no detrimental degradation products while promoting quality tissue integration and triggering minimal immunogenicity.

Nevertheless, we are excited by the fact that our understanding of tissue‐biomaterial interplay is growing, insightful new discoveries into the biology of wound healing and chronic wounds are being continually uncovered around the world,^[s41‐s51]^ and progress is being made on our ability to design materials that interact with biology over time in a bidirectional way. With help from regulatory bodies to ease advancement of promising combination therapies for a medical condition with worse survival rates than some cancers, we are optimistic that new interventions will soon be developed that will have a meaningful effect on reducing the devastating impact chronic wounds have on society.

## Conflict of Interests

The authors have declared no conflicting interests.

## Author Contributions

All authors contributed to the conception and writing of this article.

## Supporting information


**Data S1** Supplementary References.
