# Supplementary material for: Biomaterials: A potential pathway to healing chronic wounds?
Source: Exp Dermatol. 2017 Feb 14;26(9):760–3. doi: 10.1111/exd.13290 (PMC5500184; doi:10.1111/exd.13290)
Supplement: Supplementary file 1 — Data S1 Supplementary References. [file EXD-26-760-s001.docx]

**Supplementary References**

s1. Mekkes JR, Loots MAM, Van Der Wal AC *et al*. Causes, investigation and treatment of leg ulceration. *Br J Dermatol.* 2003; **148**: 388–401.

s2. Kerr M. Foot Care for People with Diabetes : The Economic Case for Change. *NHS Diabetes Rep.* 2012.

s3. Baquerizo Nole KL, Yim E, Van Driessche F *et al.* Wound research funding from alternative sources of federal funds in 2012. *Wound Repair Regen.* 2014; **22**: 295–300.

s4. Jones JE, Robinson J, Barr W *et al.* Impact of exudate and odour from chronic venous leg ulceration. *Nurs Stand.* 2008; **22**: 53–61.

s5. Cole-King A, Harding KG. Psychological factors and delayed healing in chronic wounds. *Psychosom Med.* 2001; **63**: 216–220.

s6. Armstrong DG, Kanda VA, Lavery LA *et al.* Mind the gap: Disparity between research funding and costs of care for diabetic foot ulcers. *Diabetes Care.* 2013; **36**: 1815–1817.

s7. Armstrong DG, Cohen K, Courric S *et al*. Diabetic foot ulcers and vascular insufficiency: our population has changed, but our methods have not. *J Diabetes Sci Technol.* 2011; **5**: 1591–1595.

s8. Alavi A, Sibbald RG, Mayer D *et al.* Diabetic foot ulcers: Part II. Management. *J Am Acad Dermatol.* 2014; **70**: 21.e1-24.

s9. Blakytny R, Jude E. The molecular biology of chronic wounds and delayed healing in diabetes. *Diabet Med.* 2006; **23**: 594–608.

s10. Loots MAM, Kenter SB, Au FL *et al.* Fibroblasts derived from chronic diabetic ulcers differ in their response to stimulation with EGF, IGF-I, bFGF and PDGF-AB compared to controls. *Eur J Cell Biol.* 2002*;* **81**: 153–160.

s11. Loots MAM, Lamme EN, Mekkes JR *et al.* Cultured fibroblasts from chronic diabetic wounds on the lower extremity (non-insulin-dependent diabetes mellitus) show disturbed proliferation. *Arch Dermatol Res.* 1999; **291**: 93–99.

s12. Loots MA, Lamme EN, Zeegelaar J *et al.* Differences in cellular infiltrate and extracellular matrix of chronic diabetic and venous ulcers versus acute wounds. *J Invest Dermatol.* 1998; **111**: 850–857.

s13. Rayment EA, Upton Z, Shooter GK. Increased matrix metalloproteinase-9 (MMP-9) activity observed in chronic wound fluid is related to the clinical severity of the ulcer. *Br J Dermatol.* 2008; **158**: 951–961.

s14. Liu Y, Min D, Bolton T *et al.* Increased Matrix Metalloproteinase-9 Predicts Poor Wound Healing in Diabetic Foot Ulcers. *Diabetes Care.* 2009; **32**: 117–119.

s15. Rafehi H, El-Osta A, Karagiannis TC. Genetic and epigenetic events in diabetic wound healing. *Int Wound J.* 2011; **8**: 12–21.

s16. Martino MM, Briquez PS, Ranga A *et al.* Heparin-binding domain of fibrin(ogen) binds growth factors and promotes tissue repair when incorporated within a synthetic matrix. *Proc Natl Acad Sci U S A.* 2013; **110**: 4563–4568.

s17. Johnson NR, Wang Y. Controlled delivery of heparin-binding EGF-like growth factor yields fast and comprehensive wound healing. *J Control Release.* 2013; **166**: 124–129.

s18. Gil ES, Panilaitis B, Bellas E *et al.* Functionalized Silk Biomaterials for Wound Healing. *Adv Healthc Mater.* 2013; **2**: 206–217.

s19. Choi JS, Choi SH, Yoo HS*.* Coaxial electrospun nanofibers for treatment of diabetic ulcers with binary release of multiple growth factors. *J Mater Chem.* 2011; **21**: 5258-5267.

s20. Moura LIF, Dias AMA, Carvalho E *et al.* Recent advances on the development of wound dressings for diabetic foot ulcer treatment - A review. *Acta Biomater.* 2013; **9**: 7093–7114.

s21. Gainza G, Villullas S, Pedraz JL *et al.* Advances in drug delivery systems (DDSs) to release growth factors for wound healing and skin regeneration. *Nanomedicine.* 2015; **11**: 1551–1573.

s22. Lai H, Kuan C, Wu H *et al.* Tailored design of electrospun composite nanofibers with staged release of multiple angiogenic growth factors for chronic wound healing. *Acta Biomater.* 2014*;* **10**: 4156–4166.

s23. Pashuck E, Stevens M. Designing regenerative biomaterial therapies for the clinic. *Sci Transl Med.* 2012; **4**: 160sr4.

s24. Rice JJ, Martino MM, De Laporte L *et al.* Engineering the regenerative microenvironment with biomaterials. *Adv Healthc Mater.* 2013; **2**: 57–71.

s25. Zhong SP, Zhang YZ, Lim CT*.* Tissue scaffolds for skin wound healing and dermal reconstruction. *Wiley Interdisciplinary Reviews: Nanomedicine and Nanobiotechnology.* 2010; **2**: 510–525.

s26. Turner NJ, Badylak SF. The Use of Biologic Scaffolds in the Treatment of Chronic Nonhealing Wounds. *Adv Wound Care.* 2015; **4**: 490–500.

s27. Chattopadhyay S, Raines RT. Review collagen-based biomaterials for wound healing. *Biopolymers.* 2014; **101**: 821–833.

s28. Dickinson LE, Gerecht S. Engineered Biopolymeric Scaffolds for Chronic Wound Healing. *Front Physiol.* 2016; **7**: 341.

s29. Chan BP, Leong KW. Scaffolding in tissue engineering: General approaches and tissue-specific considerations. *Eur Spine J.* 2008; **17**: 467-479.

s30. Shen Y-I, Song H-H G, Papa A *et al.* Acellular Hydrogels for Regenerative Burn Wound Healing : Translation from a Porcine Model. *J Invest Dermatol.* 2015; **135**: 2519–2529.

s31. Armstrong DG, Lavery LA, Kimbriel HR *et al.* Activity patterns of patients with diabetic foot ulceration: patients with active ulceration may not adhere to a standard pressure off-loading regimen. *Diabetes Care.* 2003; **26**: 2595–2597.

s32. Armstrong DG, Lavery LA, Wu S *et al*. Evaluation of Removable and Irremovable Cast Walkers in the Healing of Diabetic Foot Wounds: A randomized controlled trial. *Diabetes Care.* 2005; **28**: 551–554.

s33. Hammond PT. Building biomedical materials layer-by-layer. *Mater Today.* 2012; **15**: 196–206.

s34. Chan RK, Liu PH, Pietramaggiori G *et al.* Effect of recombinant platelet-derived growth factor (Regranex) on wound closure in genetically diabetic mice. *J Burn Care Res.* 2006; **27**: 202–205.

s35. Nunan R, Campbell J, Mori R *et al.* Ephrin-Bs Drive Junctional Downregulation and Actin Stress Fiber Disassembly to Enable Wound Re-epithelialization. *Cell Rep.* 2015; **13**: 1380–1395.

s36. Khamaisi M, Katagiri S, Keenan H *et al.* PKCδ inhibition normalizes the wound-healing capacity of diabetic human fibroblasts. *J Clin Invest.* 2016; **126**: 837-853. doi:10.1172/JCI82788

s37. Zhu Y, Hoshi R, Chen S *et al.* Sustained release of stromal cell derived factor-1 from an antioxidant thermoresponsive hydrogel enhances dermal wound healing in diabetes. *J Control Release.* 2016; **238**: 114–122.

s38. Nassiri S, Zakeri I, Weingarten MS *et al.* Relative Expression of Proinflammatory and Antiinflammatory Genes Reveals Differences between Healing and Nonhealing Human Chronic Diabetic Foot Ulcers. *J Invest Dermatol.* 2015; **135**: 1700-1703.

s39. Quinn KP, Leal EC, Tellechea A *et al.* Diabetic wounds exhibit distinct microstructural and metabolic heterogeneity through label-free multiphoton microscopy. *J Invest Dermatol.* 2016; **136**: 342–344.

s40. Hedberg EL, Kroese-Deutman HC, Shih CK *et al.* In vivo degradation of porous poly(propylene fumarate)/poly(DL-lactic-co- glycolic acid) composite scaffolds. *Biomaterials.* 2005; **26**: 4616–4623.

s41. Fujiwara T, Dusher D, Rustard KC *et al.* Extracellular superoxide dismutase deficiency impairs wound healing in advanced age by reducing neovascularization and fibroblast function. *Exp Dermatol.* 2016; **25**: 206–211.

s42. Brun C, Jean-Louis F, Oddos T *et al.* Phenotypic and functional changes in dermal primary fibroblasts isolated from intrinsically aged human skin. *Exp Dermatol.* 2016; **25**: 113–119.

s43. Chang HM, Huang WY, Lin SJ *et al.* ABCG2 deficiency in skin impairs re-epithelialization in cutaneous wound healing. *Exp Dermatol.* 2016; **25**: 355-361.

s44. Thandavarayan RA, Garikipati VN, Joladarashi D *et al.* Sirtuin-6 deficiency exacerbates diabetes-induced impairment of wound healing. *Exp Dermatol.* 2015; **24**: 773–778.

s45. Huang SM, Wu CS, Chao D *et al.* High-glucose-cultivated peripheral blood mononuclear cells impaired keratinocyte function via reduced IL-22 expression: implications on impaired diabetic wound healing. *Exp Dermatol.* 2015; **24**: 639–641.

s46. Emmerson E, Rando G, Meda C *et al.* Estrogen receptor-mediated signalling in female mice is locally activated in response to wounding. *Mol Cell Endocrinol.* 2013; **375**: 149–156.

s47. Garcin CL, Huttner KM, Kirby N *et al.* The Ectodysplasin A pathway contributes to human and murine skin repair. *J Invest Dermatol.* 2016; **136**: 1022-1030.

s48. Crompton R, Williams H, Ansell D *et al.* Oestrogen promotes healing in a bacterial LPS model of delayed cutaneous wound repair. *Lab Investig.* 2016; **96**: 439-449.

s49. Turner CT, Waters JM, Jackson JE *et al.* Fibroblast-specific upregulation of Flightless I impairs wound healing. *Exp Dermatol.* 2015; **24**: 692–697.

s50. Bekeschus S, Schmidt A, Napp M *et al.* Distinct cytokine and chemokine patterns in chronic diabetic ulcers and acute wounds. *Exp Dermatol.* 2016; doi:10.1111/exd.13215

s51. Wu Y, Zhong JL, Hou N *et al.* MicroRNA Let-7b inhibits keratinocyte migration in cutaneous wound healing by targeting IGF2BP2. *Exp Dermatol.* 2016; doi:10.1111/exd.13164
